# Building a responsible innovation toolkit as project legacy

**DOI:** 10.3389/frma.2023.1112106

**Published:** 2023-03-13

**Authors:** Bernd Carsten Stahl, Lise Bitsch

**Affiliations:** ^1^Centre for Computing and Social Responsibility, De Montfort University, Leicester, United Kingdom; ^2^School of Computer Science, University of Nottingham, Nottingham, United Kingdom; ^3^The Danish Board of Technology Foundation, Copenhagen, Denmark

**Keywords:** responsible research and innovation (RRI), responsible innovation, toolkit, Human Brain Project, legacy

## Abstract

This article explores whether and in what way it is possible to employ toolkits for responsible research and innovation (RRI toolkits) as mechanisms for ensuring the legacy of RRI in research projects. Based on a review of the concept of responsible research and innovation as well as existing toolkits in the area, the article offers an account of the development of an RRI toolkit in the context of the EU- funded Human Brain Project. This toolkit is designed to integrate insights and practices of responsible research and innovation developed over a 10 year period into the project legacy, the EBRAINS research infrastructure. The article suggests that toolkits have the potential to contribute to ensuring a long- lasting legacy of work undertaken in responsible research and innovation, but that this potential requires further support from institutions and the broader research environment to become realized.

## 1. Introduction

Responsible (research and) innovation (RRI/RI), defined as the attempt to ensure acceptability, desirability and sustainability of processes and outcomes of research and innovation activities, has developed a range of activities, processes, and principles (Declich et al., 2022). These are meant to achieve the different aims that collectively constitute the greater aims of acceptability, desirability, and sustainability (Von Schomberg, 2013). Since the start of the RRI discourse there has been a lively debate on how RRI can be promoted and achieved. This covers different levels of action which range from responsibilities of the individual researcher to research projects, institutions, funding structures and broader research and innovation policy contexts. The level of the individual research project is probably the one for which there is the largest amount empirical details and insights concerning the practice and implementation of RRI (Gurzawska et al., 2017; Ryan et al., 2021). This is likely to be caused by the fact that most RRI funding has been dispersed through research projects, giving RRI practitioners ample opportunity to observe RRI integration and publish about it.

One challenge that RRI faces at the project level is that of securing a legacy at the end of the project. This challenge is not unique to RRI, as research activities and technical achievements similarly struggle to survive the end of the project and achieve lasting social (Abelson and Gauvin, 2006) as well as technical or organizational impact (Briggle, 2019; Donovan, 2019). Unlike in the case of scientific and technical work, however, there are no established legacy mechanisms for RRI, such as follow-on project funding or market-oriented venues, such as spin-off companies, venture capital, etc. (Scholten and Duin, 2015). This paper reports on the efforts of achieving a project based RRI legacy by creating a toolkit that is meant to persist beyond the project funding. We believe that this may be a viable, if partial, avenue to secure longer term project impact and legacy.

We illustrate this point using the example of the EU Future and Emerging Technologies Flagship Human Brain Project (HBP), has invested heavily in RRI-related activities since 2013. The project ends in October 2023. A key focus of the work leading up to the project's conclusion was to develop and assemble an RRI toolkit that builds on the various strands of this work. The toolkit is designed with the aim of supporting activities in EBRAINS. The EBRAINS research infrastructure being the main legacy mechanism of the HBP. The paper elaborates on the process of conceptualizing the EBRAINS RRI toolkit and implementing it. It outlines the content of the toolkit and how it relates to prior RRI work. Our overarching research interest is in finding out how a toolkit supports the legacy of RRI project work. As the work within the HBP and EBRAINS is ongoing at the time of writing we cannot offer a final answer to this question and therefore focus on the more specific research question of: “how can an RRI toolkit be constructed with a view to supporting RRI legacy.” By exploring this question and discussing our approach, the paper offers a space for reflection on the disadvantages, downsides and pitfalls of using the concept of toolkit as means to secure RRI legacy.

As the paper reports on an ongoing activity, we cannot provide final answers to the research question. We can, however, draw on our work and observe similar past developments to identify key challenges. We contend that RRI toolkits do offer the opportunity to shape the legacy of RRI activities on a project level, but they are unlikely to remain impactful, if abandoned or left to their own devices. RRI toolkits need to hook into other legacy mechanisms to ensure that resources are available to build the capacity to use them, provide motivations for doing so and to maintain their content and level of relevance. Very briefly, RRI toolkits can thus only form a partial answer to the problem of RRI legacy. We believe that this insight is conceptually interesting because it goes to a key problem of the RRI discourse that remains unaddressed. If RRI is about changing practices, then a legacy that is based exclusively on academic publications is not satisfactory. So far, however, there has been little literature on how a legacy beyond publications can be achieved. The article is therefore of interest to the members of the RRI research community, but also speaks to scholars who may not consider themselves part of this community but who have a role in implementing and realizing RRI. The article is furthermore of relevance to other stakeholders and decision makers in the research and innovation environment who are considering embedding and promoting RRI and who may need to consider the wider question around RRI legacy.

The paper progresses as follows. We provide an introduction to the RRI discussion and in particular the question of RRI project legacy. This includes the specifics of our case example of the HB and how legacy was planned in this project. We then describe the development and content of the HBP RRI toolkit, drawing on insights from other projects that have taken similar approaches. This provides the basis for a critical discussion of the idea of RRI toolkits as a legacy mechanism. We explore the limitations of our approach and theoretical as well as practical implications for researchers and the wider research environment.

## 2. RRI and the question of its legacy

This section sets the scene by offering a brief introduction to the concept and definition of RI/RRI. This provides the backdrop for the introduction of the description of our RRI activities in the Human Brain Project. Our current challenge, the question of the post-project legacy, will be introduced at the end of the section.

### 2.1. Responsible (research and) innovation

The concept of responsible research and innovation has been discussed since the early 2010's (Von Schomberg, 2011; Stilgoe et al., 2013). There is an entire stream of publications dedicated to the concept (Wiarda et al., 2021), its intricacies and limitations and, not least, to the difference between responsible innovation and responsible research and innovation (Owen and Pansera, 2019; Owen et al., 2021). We assume that readers of this paper are at least broadly familiar with the concepts and do not wish to belabor them unnecessarily. However, we need to clarify our working definitions to ensure the academic integrity of the article.

We do not see a major difference between responsible innovation and responsible research and innovation, even though we are well aware of the discussion of the terms. We believe that von Schomberg's definition that we alluded to in the introduction is compatible with definitions from other streams of the RRI discourse:

“Responsible Research and Innovation is a transparent, interactive process by which societal actors and innovators become mutually responsive to each other with a view to the (ethical) acceptability, sustainability and societal desirability of the innovation process and its marketable products (in order to allow a proper embedding of scientific and technological advances in our society).” (Von Schomberg, 2013, p. 63)

We believe this to be consistent with the other dominant positions, notably the one by Stilgoe et al. (2013) that defines RI as “taking care of the future through collective stewardship of science and innovation in the present,” covering the four dimensions of anticipation, reflexivity, inclusion and responsiveness. This, in turn, is consistent with the European Union's definition of RRI as “the on-going process of aligning research and innovation to the values, needs and expectations of society” (Rome Declaration, 2014).

RI/RRI as a concept came to prominence in the early 2010's. It has much older roots, however, drawing on prior discourses and research streams in areas such as Science and Technology Studies (Owen, 2013), philosophy of technology (Dusek, 2006), or technology ethics (Brey, 2011). One particular strain of work that fed into RI/RRI is that of ELSI (ethical, legal, and social implications). Some scholars view ELSI as closely related and similar to RI/RRI (Komiya et al., 2022) whereas others view it as a distinct predecessor which has prepared the grounds for RI/RRI as a post-ELSI activity (Balmer et al., 2016). For the purposes of this paper these conceptual distinctions are of secondary importance and we can leave their discussion to the relevant expert discourses. For us the questions of implementation are of relevance as well as the question of which challenges for the longer-term viability of the resulting activities arise. For the purpose of simplicity, we will henceforth use the term RRI which was dominant in our work, but with a clear caveat that we believe this covers the RI discussion as well.

### 2.2. RRI in the Human Brain Project

The Human Brain Project (HBP) is a European research project that was funded under the Future and Emerging Technologies Flagship funding scheme. Only three such projects were ever funded. The idea behind these flagship projects was to provide large amounts of resources to large and multidisciplinary consortia that would have the opportunity to make significant progress in their area of research. In the case of the HBP this translates into a consortium including more than 100 partner organizations, a duration of 10 years (from 2013 to 2023) and a core budget of more than €400 million. This was to be complemented by national and other sources of funding.

On a content level, the HBP brings together neuroscience and ICT / computer science to work on a range of topics of shared interest. This includes data curation, storage and management, provision and sharing of neuroscientific insights e.g., in the form of brain atlases, brain simulation as well as the development of novel ICT tools and paradigms such as neuromorphic computing or neuro-robotics.

The internal interpretation and external perception of the project have changed over time. Following some high-profile interventions early in the project (Frégnac and Laurent, 2014) and extensive public debate, the project reformed its leadership structure (Abbott, 2015) and clarified its scientific direction (Amunts et al., 2016). The focus of the project has since been very clearly on the development of an ICT research infrastructure for neuroscience.

By bringing together research from neuroscience and ICT, the HBP always raised the potential of leading to broader ethical and social concerns deriving from both fields. As a consequence, the project included a strong component on RRI from its outset (Rose, 2014). The activities pertaining to this were initially part of a sub-project on ethics and society and now, during the last 3-year period are bundled in a work package on RRI but can also be found distributed across the scientific and technical sections of the project.

The RRI-related work of the HBP was planned and implemented as consisting of research on RRI and the various topics covered by it in the project as well as the provision of services related to RRI, such as ethics management and data protection support. The RRI-related research undertaken within the project has led to a string of publications covering attempts to identify and categorize the relevant issues (Christen et al., 2016) and description of the general approach and organization of RRI (Salles et al., 2019a; Stahl et al., 2019) as well as work on specific topics and issues, such as neuroethics (Evers, 2016; Salles et al., 2019b) or dual use (Ulnicane et al., 2022). We have published a number of articles aiming to provide insights into our approach to RRI and possible weaknesses and future ways forward (Aicardi et al., 2018; Mahfoud, 2021; Stahl et al., 2021; Aicardi and Mahfoud, 2022). As all this work is well-documented, we do not wish to revisit the detail but focus on the question of what will become of it after the project finishes.

### 2.3. The future of RRI in EBRAINS

The ostensible aim of the HBP at this point is the creation of a distributed European research infrastructure for neuroscientific research. This work is well-advanced. It has resulted in the formation of EBRAINS which is both the name of the infrastructure itself and of a non-profit legal entity based in Belgium, which has taken over the coordination of the HBP in 2021 and which leads and coordination of the formation of the infrastructure. EBRAINS has successfully applied to be included in the 2021 ESFRI roadmap, which confirms its status as a developing European infrastructure, and has provided momentum for its further development. In 2022 EBRAINS worked on the formalization of its governance structures and consolidation of membership. The EBRAINS membership is made up of national nodes which contribute to and support the overall infrastructure, and which consist of various organizations in the respective country that provide the services that will make up EBRAINS.

While this scientific and technical development of the EBRAINS infrastructure is on its way, one stream of work is dedicated to identifying how the insights and processes that arose from the RRI-related work can be retained and integrated into the infrastructure. For this purpose, a task force was created that was chaired by the CEO of EBRAINS and consisted of members representing different aspects of RRI as well as the scientific and administrative leadership of the project. In order to provide a baseline conceptual starting point for this work, the task force, following internal and external consultation, published an “Ethics and Society Vision.^1^” The task force furthermore deliberated on RRI priorities and ways of integrating such work into EBRAINS. This led to the definition of an EBRAINS Ethics and Society Committee which will take ownership of these issues in the EBRAINS infrastructure. At the time of writing, this task force is in the process of being set up.

A final remark on the transition from a research project to an infrastructure refers to the sources of funding. The HBP is centrally funded through established EU funding mechanisms, predominantly through the Horizon 2020 Research Framework Programme. European infrastructures tend to be funded decentrally through the Member States which provide those services that they deem to be in their national interest. In the case of the HBP, there was a specific funding call in the Horizon Europe Framework Programme on “Research infrastructure services to support health research and accelerate the digital transformation”^2^ which offered funding earmarked for the development of the EBRAINS infrastructure in the transition phase from funded project to infrastructure. It is notable that this call pays very little attention to ethical and social issues and makes no reference to RRI. As a consequence, the funding from this call can only be spent on RRI-related work to a very limited extent, thus exacerbating the question of how the HBP RRI work can be transitioned into the EBRAINS infrastructure. This is where the idea of using a toolkit as a legacy mechanism finds its justification. The implementation of the HBP toolkit is now described in the following section.

## 3. The development of the EBRAINS Ethics and Society toolkit in the HBP

Having provided the background on the concept of RRI and its implementation in the HBP, we are now in a position to elaborate on the EBRAINS RRI toolkit. We do this by discussing the concept of toolkits and reviewing some that have high prominence in the RRI space. We then elaborate on how we have approached the creation of our toolkit and its content.

### 3.1. Toolkits as support for responsible research and innovation practices

The Cambridge dictionary contains two definitions of the word toolkit. One related to the workplace as “*a set of tools that are used for making or repairing something*,” and another for the context of Human Relations, or HR, as “s*kills and knowledge that are useful for a particular purpose or activity, considered together*” (Cambridge Dictionary, 2022). Looking up the word toolkit, the definition simply states “*A set of tools*.” In our definition of the EBRAINS Responsible Research and Innovation Toolkit (EBRAINS RRI Toolkit), we define it as a collection of tools that consist of knowledge and skills for undertaking practices defined as belonging under the framework of responsible research and innovation. Furthermore, the tools we develop are meant to support “*EBRAINS users furthering reflection on the (neuro)ethical, philosophical, social, and diversity implications of their work. Moreover, the toolkit will include guides for engaging stakeholders and diverse publics, foresight exercises on possible future implications, and examples of ways to take action to address such issues” (internal working document, 2021: 5)*. The ultimate aim was that the tools would result in users that could themselves identify such issues and know how to address them. The EBRAINS users that we had in mind cover a variety from neuroscience and neurotechnology researchers, data and infrastructure providers to management and leadership level of the EBRAINS infrastructure.

The toolkit that was developed as part of the HBP is process oriented to support users in their research work, or in their use of the EBRAINS research infrastructure, by providing ways to go about all the phases of their research-related work, from formulating proposals for research or policy, to programme development, project definition, research activity and the implementation of results and innovative outcomes. It follows a long line of other projects that looked to the toolkit mechanism as a way of leaving an output that would live on beyond a limited project period. Table 1 gives an overview of some of these, and maps how they address the Framework of RRI and the European Commission keys of RRI (European Commission, 2013).

**Table 1:** Overview of RRI toolkits.

**Origin of toolkit/defining features^a^**	**RRI framework area addressed**	**EC keys addressed**	**Disciplinary/topical focus**	**Area of contribution**	**Audience**
RRI tools (https://rri-tools.eu/about-rri)	Focus on process of development that should be: Diverse and inclusive, anticipative and reflective, open and transparent, responsive and adaptive to change. Outcome requirements to achieve: Engaged publics, responsible actors, responsible institutions. Ethically acceptable, sustainable, and desirable outcomes of research and innovation, and lead to solutions to the EU “seven grand challenges”	Ethics, gender equality, governance, open access, public engagement, and science education	No specific disciplinary focus	Policy	Policy makers, research community, education community, business and industry, and civil society organizations
GoNano^b^ (http://gonano-project.eu/road-of-co-creation-training-materials-researchers-engineers/; http://gonano-project.eu/wp-content/uploads/2020/05/GoNano_Co-Creation-toolkit_DEF.pdf)	Reflection, anticipation and engagement and action through co-creation	Ethics, gender equality, public engagement	Nanotechnologies	Research	Researchers and engineers
PRISMA (Porcari et al., 2019); https://www.rriprisma.eu/rri-toolkit/	Reflection, anticipation and engagement and action	Ethics, gender equality, and public engagement	Standards for research and innovation (responsibility-by-design)	Corporate social responsibility, management, quality, research, and safety	Small or medium sized companies, innovators, and entrepreneurs
ResAgora (Lindner et al., 2016); https://responsibility-navigator.eu/navigator/	Responsiveness and dialogue	Governance	Acceptable outcomes of governance practices	Policy	Governance bodies
Action Catalog (http://actioncatalogue.eu/about)	Reflection, anticipation and engagement and action	Public engagement	Reflection, anticipation and engagement and action through co-creation	Policy and research	
Multi-act toolbox (https://toolbox.multiact.eu/)	Reflection, anticipation and engagement and action		Developing patient engagement plans with patients	Brain disorders	Pharmaceutical industry, research consortia, research institutes, health products or services companies, patient organizations, brain health-focused NGO, universities, and neurology hospital departments
Orbit self-assessment tool (Stahl, 2017); https://www.orbit-rri.org/tools/self-assessment-toolfree/	Anticipation, reflection, engagement on process of innovation and outcome	Governance, Gender, Open science, and science education	Inspire ICT developers to new practices	Computer sciences	ICT developers
KARIM (Hin, 2012; Iatridis, 2015); https://www.nweurope.eu/media/1118/guide_online.pdf	Anticipation of needs and desirability of stakeholder and focus on outcome of innovation process: sustainability (environmental, social, and economic)		Inspire responsible business development	Business innovation practices	SMEs
Responsible innovation compass (COMPASS Project, 2020)	Anticipation, reflection, engagement (of researchers) and action through co-creation	Ethics, Governance, gender equality public engagement, science education	ICT, nanotechnology, and healthcare	Business innovation practices	SMEs
EBRAINS Ethics and Society toolkit (https://ebrainsethicsandsociety.tekno.dk/)	Focused on initiating reflection with the user of the toolkit material	Ethics, gender equality, public engagement	ICT-driven Brain science	Research and policy	EBRAINS users and researchers

^a^Lehoux et al. (2020) presents the Responsible Innovation in Health Tool (RIH), and also analyze several other RRI-based tools for innovation in industry and business. Here we included the Orbit self-assessment tool, KARIM, Responsibility Navigator. Our analysis of these tools in this table is inspired by Lehoux et al. (2020). They also analyze the RMol tool developed by Long et al. (2020) and an ICT-based tool developed by Flipse (2018). These two last tools, and the RIH tool, are not included in our analysis as we could not access the tools or find shared resources for others to apply the tools.

^b^Malsch (2013) mentions three RRI tools developed within Nanotechnology and funded by the European Commission. ObservatoryNano (https://cordis.europa.eu/project/id/218528), NanoCode (https://cordis.europa.eu/project/id/244521), and EthicSchool (https://cordis.europa.eu/project/id/36745). These three tools are not included in the present analysis as we could not find a website for the projects that contained materials to read through and use for one's own practice.

Table 1 gives an overview of some of the toolkits that have been developed in the context of research projects. The table gives a reference to the origin of the toolkit, and shows what parts of the RRI framework, EU RRI keys that the frameworks address, as well as if the toolkits have been focused on particular topics or disciplines for making an impact, as well as what area of social activity the toolkits were aimed at, and who the intended recipients were.

We do not claim that this table provides a comprehensive overview of all RRI toolkits. Construction of the table followed a three-pronged approach. A first search was done in in Google Scholar using the search term “responsible research and innovation toolkit” and the search term “responsible innovation toolkit.” The first five pages of the search was reviewed to find articles with a heading that included the words “tool” or “toolkit” together with responsible research and/or innovation. The first combination of search terms yielded one article (Malsch, 2013), and the second combination of search terms yielded one article (Lehoux et al., 2020). In the second step, a general search on the terms “responsible research and innovation toolkit” was caried out using Google and the first 70 entries were reviewed for presentation of toolkit with a background in theory on RRI. The search resulted in multiple references to the RRI toolkit of the EU project RRI tools. Finally, this search was complimented with toolkits, or tools from those EU research projects we were most familiar with. Although not the sole funder of RRI, the European Commission has arguably been the largest funder over the last 10–15 years of RRI, and there we focused on EU projects.

One noticeable insight from the table is that there seem to be subject specific issues that are addressed by the tools, but there is also a common core that most of them share. One aspect that most of them have in common is an explicit focus on promoting and facilitating reflection (Grimpe et al., 2020). Our observation of the strong emphasis on reflection in various toolkits gives rise to the question why this is such a strongly recurrent theme. As outlined earlier, most accounts of RRI see reflexivity as one of several components of the concept which also includes other such as anticipation, engagement, or responsiveness. These aspects of RRI are not independent of one another as Fraaije and Flipse (2020) have shown. We speculate that one of the reason for the focus on reflexivity is that RRI as a whole can be interpreted as an attempt to integrate second order reflexivity into the research system. All research must display a certain level of reflexivity, e.g., when considering data sources, analysis methods or research implications. RRI aims to promote the reflection of this reflexivity, hence the reference to a second order reflexivity (Wynne, 1993). This is an aspect of the RRI debate that is worth pursuing further, but we will refrain from doing so here, as it would lead us too far from the subject matter of the article.

While there are thus some commonalities of the toolkits such as the focus on reflexivity or public engagement, there are also notable differences. One of these refers to the subject area of the projects, which include topics like nanotechnology and medical research. Several of them were focused on particular mechanisms to realize RRI, such as standards or organizational governance structures. As a result they target different audiences, which range from individual researchers to research policymakers and companies.

One lesson derived from exploring these existing toolkits was that, despite the shared overall aims of RRI across disciplines and levels of activity, there appears to be a need for specific and targeted tools that address the specific needs of particular users and user groups. The discourse and frameworks of RRI tend to be abstract. This in itself creates a need to operationalize abstract and theory-heavy frameworks for use in practice. RRI is directed at inspiring reflective changes in innovation processes and their outcomes. Science and research consists of many epistemic cultures (Knorr-Cetina, 1999), and engineering disciplines and technology development can be understood as guided by shared beliefs, norms and functional structures for innovating (Belt and Rip, 1987; Kemp, 1994; Rip and Kemp, 1998). It therefore becomes crucial for operationalizations of RRI to both address and challenge specific epistemic cultures and practice-bound ways of innovating. Tools building on the norms, beliefs and practices within a given field achieve the former, while exercises of anticipation, reflection and engagement aim to challenge established cultures and ways of thinking, and crate reflexivity among the community of users of those RRI tools. This insight supported our intention to develop a toolkit specifically targeted at current and potential users of the EBRAINS research infrastructure.

### 3.2. Developing the EBRAINS Ethics and Society toolkit, from plans to end result

Several key decisions were made early in the development of the EBRAINS toolkit. One of the first was that the title of the toolkit would not directly refer to the concept “RRI.” The decision was made based on the understanding that for the audience we wanted to reach, RRI would not present a familiar or catchy phrase. Instead, a decision was made to go with the more general title “Ethics and Society toolkit.”

The final toolkit was designed across a three-dimensional approach: multi-media-friendly, to encourage anticipation, reflection, to inspire to new practices, and to be expandable. Adapting to a multi-media environment meant the toolkit is built on short texts, video, using scenarios to present dilemmas, and creating a structure where links guide the user toward further engagement and knowledge. The multi-media dimension meant we developed the toolkit as a digital, online and interactive tool. It should be adapted for viewing across devices, so also adapted to smaller screens, and we decided that the toolkit should follow accessibility standards set out in the EU directive 2016/2102 (The European Parliament and the Council of the European Union, 2016), ensuring the toolkit can be accessed with screen readers, and that any graphics use contrast and colors that works also for the visually impaired (including color vision impairments).

With regards to the RRI dimension, the focus is inspiring reflection, anticipation, and inspire the user. Reflection is encouraged by confronting the user with an alternative perspective of their own practices to inspire reflection on related ethical and social issues. Anticipation is supported through the inclusion of foresight elements like scenarios. Expandability refers to the use of a linking structure, to guide the user on consecutive steps of reflection. Finally, we added a self-assessment quiz, to support reflexivity and learning. The quiz became the solution to initiating user reflection and learning, since, users will most likely engage with the toolkit without any guidance. Finally, the toolkit was also designed in line with the EBRAINS design layout, color scheme, and compatible with the content management system in the EBRAINS.eu web portal. This was done to strengthen the connection with EBRAINS and our aim of using the toolkit as a vehicle for our legacy.

In addition, a key design choice was to tailor the EBRAINS RRI toolkit to the neuroscience community and to EBRAINS users. Each selected theme in the toolkit is therefore focused especially on neuroscience themes and cases. The frontpage of the toolkit contains a message that underlines the relevance and specificity of the tool to EBRAINS users. As with all resources on RRI, the toolkit is not meant as a checklist for the user to make sure they are responsible. Rather the design and infrastructure of the toolkit should make the user more aware of their own reflections and biases, open for further debate within their team and guide the user through planning and taking action.

Furthermore, as the toolkit is developed as a legacy mechanism for our RRI related work in the HBP, it builds on previous work in the HBP. Therefore, it is connected with an extensive package of training and capacity building modules, that was also developed to support future EBRAINS user. In the final presentation of the toolkit, entry points ended up being thematic, allowing easy access for users with no prior knowledge of RRI. Researchers are thus guided on the basis of the content that would be most immediately relevant. When users engage with one tool in the toolkit, they are encouraged to visit other topics and tools.^3^

### 3.3. Content and process for integrating the toolkit in EBRAINS

In this section we go a bit deeper into the content of the toolkit. We also expand on the process of negotiation to integrate the toolkit in the EBRAINS research infrastructure. The EBRAINS Ethics and Society toolkit builds on the work done under the framework of RRI, philosophy and neuroethics in the HBP. Topics for the toolkit were invited from all collaborators in the project and we ended with a list of four topics: Public Engagement, Data Governance, Equality, Diversity and Inclusion, and Neuroethics. The topics are not definitive as the toolkit is designed so that EBRAINS can assess, change, adapt and further develop it after the end of the HBP. The toolkit is developed as an avenue for exploration of how to work on and ensure legacy of RRI in the transition from a research project to a research infrastructure. The toolkit is therefore not a finished product, but developed as the starting point of a learning and capacity building environment. A limitation of the present toolkit is its status as an outcome of negotiation and collaboration within an interdisciplinary group. Even if there is agreement on the overall need to simplify the presentation of topics and of RRI, the actual way in which such simplification should be done was not a straightforward process. Early on, the group leading the development of the toolkit decided on a general format of presentation for all topics. For example the format require a video presentation of the anticipatory foresight element for all contributed tools. As a consequence some collaboration partners withdrew from the collaboration as the frame was experienced as narrow and too general. These types of negotiations are typical of interdisciplinary collaboration [see also Stahl et al. (2021), where we describe some tensions we experienced in interdisciplinary work]. Ultimately, consideration of user needs for easy access and recognizability pushed the decision for a general format across all topics.

**Figure 1:**
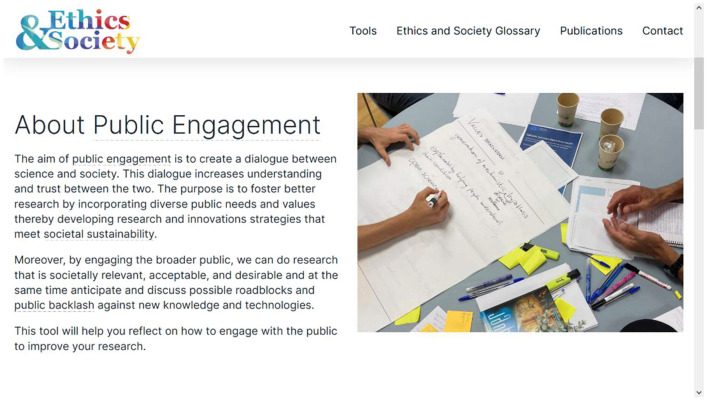
Introduction to public engagement in the EBRAINS Ethics and Society toolkit.

Each topical presentation follows the same logic. A short introductory text, followed by a presentation of a foresight scenario-based dilemma. Figure 1 shows the topical introduction of the public engagement element of the toolkit. The description very briefly introduces public engagement as a way of steering research toward societal benefit, before directing the user to the resource that will help them reflect on how to engage with different publics in their work.

**Figure 2:**
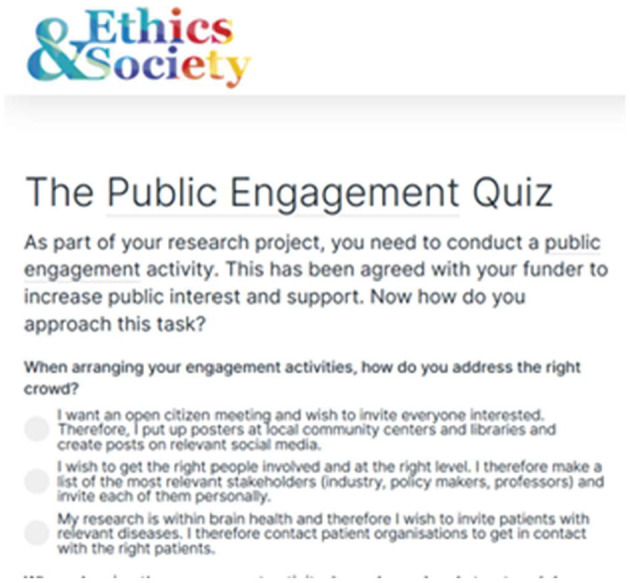
A snapshot of the quiz for public engagement in the EBRAINS Ethics and Society toolkit.

In the next step, user reflection is initiated through a quiz (see Figure 2). In the quiz none of the answers are wrong answers. Instead the answers present different options for actions to encourage the user to reflect on own practices and realize there are several ways of engaging with an ethical or social issue. For the opportunity to further explore the topic and the RRI background, the user then is offered links to relevant academic publications on the topic produced in HBP, the capacity building and teaching material developed in the HBP as another legacy measure, and links to the EBRAINS Community Space.

For the foresight element of the toolkit. Each video for each topic follows a similar outline to create consistency and coherence across the toolkit and presentation. Each video is kept short to encourage online sharing and increase the chances that the full video is viewed. The framework for each video follow the outline:

“*In a future to come, the human brain is now better understood than ever. [Main character: researcher/research team/PhD student/staff] are leading researchers within the field of [insert relevant research field]. The team hope that [future positive aim] and has just [new event]. However, [insert turn of events with relevance for the topic]. Now [main character] is considering how to best [incorporate topic relevant reflections]. [Insert dilemma and reflection questions]. What would you do if you were [main character]? Learn more about how these questions can be met by interacting with the tools about [topic] on this page (Bitsch et al., forthcoming).”*

**Figure 3:**
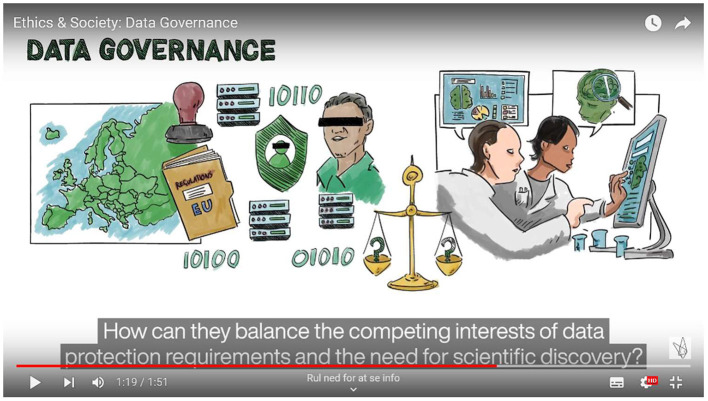
Screenshot of the foresight element in the EBRAINS Ethics and Society toolkit for data governance.

Each of the videos ends with questions for the user to reflect upon the questions focus on a topic-based dilemma. The users are asked how the dilemma might reflect on their own practices. Figure 3 below shows the second question asked on data governance “How can they balance the competing interests of data protection requirements and the need for scientific discovery?,” this question is preceded by “Considering the available regulations in Europe how can they best protect their data subjects?,” and followed up by the question “What policies and procedures should the researchers implement to protect their data subjects?” Data governance is one of the topics that has been a concern of the research team since the beginning of the HBP. The questions build on experience coming from interacting with researchers in the HBP. They address users' frequently asked questions, but also challenges them to look for ways of balancing their own interests with that of society or data subjects.

At present, there are no clear agreements with EBRAINS concerning the incorporation of the toolkit on the EBRAINS website. Several approaches were taken to find a way toward integration of the toolkit. One avenue was to style the toolkit as a “service.” EBRAINS offers a number of services, and the toolkit could fit this category. However, in conversations with EBRAINS, we realize that the services they would like to present include purely technical and platform based ones. Presently, options for integration could come from the adaption of an EBRAINS vision on Ethics and Society (EBRAINS, 2022). This high-level vision includes many statements that could justify offering practical tools for the infrastructure users.

## 4. Reflections on the EBRAINS Ethics and Society toolkit

We have framed our creation and implementation of the EBRAINS RRI toolkit in terms of provision of project legacy and post-project relevance of the work we have undertaken. The mix of motivations and intentions that led colleagues to contribute to the development of the toolkit is of course in practice much broader than the specific focus on legacy. Reasons for turning prior research and other insights into the project structures into a set of tools are likely to include a range of motivations from an attempt to secure the visibility of the contributions of individuals and groups, all the way to the enjoyment of undertaking creative work and building novel artifacts. This discussion of the HBP RRI toolkit does not take into account these broad sets of motivations but focuses on the question of RRI legacy.

In order to understand the potential future role of the toolkit, it is helpful to consider current expectations of its future use. The intention behind including a toolkit in the plan for the final phase of the HBP was to provide the means for future users of the EBRAINS research infrastructure to find ways of appropriately dealing with the ethical and social issues that their work may raise. The expectation is that, at least initially, users of the infrastructure will include a high percentage of current HBP members or users that are already enrolled during the project phase. This is the reason why, in addition to the development of a toolkit, there was strong emphasis on capacity building. In practice this included a range of activities, most of which in the form of training sessions which covered all aspects of the RRI work undertaken in the project. Many of these directly fed into individual tools in the toolkit. The hope behind this approach is that the users who have developed relevant capacity to understand and deal with the issues are going to be the first generation of tool users who can put the tools in practice in their research activities.

When compared to other RRI toolkits, it is clear that our toolkit has a specific focus on the interaction of neuroscience and ICT. Some of the toolkits we reviewed earlier aim to appeal to a broader audience and offer general purpose tools for researchers. This is not the case for our toolkit that was specifically designed for the users of the EBRAINS infrastructure and thus presupposes a particular set of user backgrounds, skills, and prior experiences.

While this design of the toolkit for a specific scientific community should hopefully be plausible, it should also be clear that the pathways to achieve this vision or its exact details are at present not determined. This has to do with the uncertainty of the exact shape of the EBRAINS infrastructure itself. A key difference between the HBP and EBRAINS is that the HBP as a research project has a centralized funding structure which requires a certain type of governance. This will change in the transition to the infrastructure. The EBRAINS infrastructure will be distributed and consist of national nodes which have institutions in their countries as members. These national nodes will collectively constitute the infrastructure and decide which services will be provided. Importantly, most of the funding for the infrastructure will come directly to the national nodes which will change the governance structure and most likely the power dynamics. The EU provides some additional funding for the further development of the infrastructure as a grant under the research framework programme Horizon Europe, but the resources that are centrally available will be significantly less than is the case under the project. One consequence of this changing structure is that the exact detail of the services that EBRAINS will provide depend on national funding and is subject to change. This clearly has implications for the type and scale of ethical and social issues that may arise from the service and thus to the type of RRI-related activities that need to be provided. This future uncertainty has implications for the RRI toolkit which is based on current needs and requirements which may well change based on changes in the services provided and thus the nature of the EBRAINS infrastructure. To put it differently, the tools being developed are responses to the current problems but there is no guarantee that they will remain relevant to tomorrow's problems.

This is an important first point in a more general critical reflection of our approach to developing an RRI toolkit to ensure our work's legacy. Our research provided an answer to the research question: “how can an RRI toolkit be constructed with a view to supporting RRI legacy.” The account of the development of the toolkit described earlier will hopefully be of interest to others who are tasked with developing such toolkits. The broader context of this question, however, was the role of a toolkit with regards to supporting RRI legacy. This is not a question we can factually answer as the project is still ongoing, and a proper assessment of the legacy will only be possible sometime after the end of the project. However, our current position and insights allow us to offer some critical reflections.

The development of the toolkit took place in parallel and often based on activities to develop capacity of current HBP members and EBRAINS users. Anecdotal evidence suggests that the take-up of the capacity building activities, most of which took the form of online training courses, was not overwhelming. Even where strong numbers of applicants to attend capacity development activities were received, the actual turn-out was typically <½ of the number of applications. This may have to do with “zoom-fatigue,” the fact that after the COVID-19-related lockdown and enforced collaboration *via* electronic means, many people no longer wished to engage in online interactions. What it indicates for the toolkit, however, is that one should not expect huge numbers of people to engage with it. A key consideration therefore must be, in addition of questions of usability discussed earlier, the question of motivations for individuals to use the tools. This links directly to processes and governance of the EBRAINS research infrastructure. If certain tool uses are mandated as part of the access requirement, this may make users engage. At the same time, such mandates would increase the cost of usage of the infrastructure and might thus discourage some users, which the infrastructure would like to avoid. The question of the integration of the toolkit as the legacy of RRI into EBRAINS as the legacy of the HBP is thus crucial, but at present not resolved.

A related question is that of the responsibility for the toolkit. We have already alluded to the fact that the toolkit will need to be updated and kept current. This has a content component, but it also has an organizational and governance component. For the content to remain current, someone has to be responsible for its maintenance. This requires resources as well as workflows that would flag outdated content, identify new content and an approval process for new content to replace old content. These resources and workflows are currently not confirmed.

Our approach as recounted here has several limitations, most of which we have previously alluded to. We have provided an account of how such a toolkit can be created. However, we are presenting our account of the toolkit during the lifetime of the project, which precludes us from providing data on post-project usage and uptake. Moreover, we are reporting from our experience in one project, which in addition is a very special case and does not lend itself to drawing more general conclusions with immediate applicability to other projects.

While conceding these limitations, we still think that the article will provide insights that are relevant to both the RRI research community and more broadly to scholars, funders, research managers or policymakers with an interest in RRI. The HBP does not provide a generalizable basis in terms of statistical significance, but it is a high-profile science project whose RRI-related activities have been extensively reported and that can be seen as a public laboratory for realizing RRI in practice.

One central insight into the nature of RRI that this article confirms is that it must be a multi-level practice, if it is to be successful. This means that project-level activities are important. This refers to the work of researchers and the structure and governance of a project where RRI can be implemented. The creation of an RRI toolkit can form part of these activities. It is abundantly clear, however, that the eventual success of these activities beyond the immediate confines of the project depend on factors outside of the project's control. As our example has shown, the eventual location and usage of such a toolkit depends on organizational policies, in our case this includes EBRAINS, but it will often also be universities or other organizations where research is undertaken. These, in turn, are reactive to signals sent by funders and research policy.

A lack of policy support is thus a significant disadvantage for the successful uptake of an RRI toolkit. In our case, the funding call for the further development of the EBRAINS research infrastructure contained no requirements for RRI integration which made it more difficult to find a way to embed the toolkit in a sustainable manner. More broadly, the attention paid to RRI on the European level appears to be decreasing. After strong emphasis on RRI in the Horizon 2020 research framework which ended in 2020, there is much less visibility of the term in its successor, the Horizon Europe framework programme. While there is still significant emphasis on aspects of RRI, such as gender equality or research ethics, the overall term is receiving less attention. This was one of the reasons why our RRI toolkit ended up without including the RRI acronym and was instead called Ethics and Society toolkit.

A further open question is whether RRI toolkits can be successfully divorced from their originators and still remain usable and relevant. RRI is not a simple and straightforward matter, neither conceptually nor empirically. It is therefore not clear whether a toolkit can have the desired impact as a stand-alone resource that potential users can access and utilize. This, to a large extent, is an empirical question that will depend on the quality of the toolkit, but also on the topic, audience and content. Anecdotal evidence suggests that the separation of the tool from its originators poses non-trivial challenges. So far we are not aware of any usage statistics of existing toolkits such as the ones listed in Table 1. We therefore have no data to assess whether RRI toolkits are used and whether this use is successful.

This points to the question of the broader context of the toolkit within the RRI discourse and the research ecosystem. We continue to believe that a toolkit can provide a useful mechanism for the transmission of RRI insights into post-project practices. However, these reflections also indicate that the existence of an RRI toolkit may be a necessary condition for successful legacy, but it is by no means sufficient. In order for the RRI toolkit to gain and maintain relevance, several other components need to be available in the research ecosystem.

## 5. Conclusion

In this article we have looked at the idea of RRI toolkits and suggested that they may fulfill the role of a project legacy. Based on a conceptual analysis and our experience in the HBP we have then described how we have designed and developed our RRI toolkit and how this is to be integrated into the overall project legacy, the EBRAINS research infrastructure.

In answer to our research question we have provided an account of the creation of the HBP RRI toolkit that is based on a decade of RRI research in the project. This is an important contribution to knowledge in that it gives an example of how the requirements informing the toolkit design can be translated into the practice of toolkit development. The question that the paper cannot answer pertains to the broader context of securing RRI legacy post-project. Giving such an answer would require empirical investigations of real toolkits post-project as well as a more detailed analysis of legacy which points to complex concepts such as impact that would need to be unraveled in the context of RRI. Such a broader analysis that would need to include other stakeholders such as research performing organizations, research funders and policymakers will be required to determine RRI toolkits such as the one described here are suitable mechanisms for achieving RRI legacy. We hope that this article can serve as the basis for such future research.

## Data availability statement

The original contributions presented in the study are included in the article/supplementary material, further inquiries can be directed to the corresponding author.

## Author contributions

BS led the structure and drafting of the article. LB led the analysis and description of the RRI toolkits and the EBRAINS Ethics and Society toolkit. Both authors contributed to discussion and conclusion. Both authors contributed to the article and approved the submitted version.

## Footnotes

^1^https://ebrains.eu/ethics-and-society-vision/, accessed 10.10.2022.

^2^https://ec.europa.eu/info/funding-tenders/opportunities/portal/screen/opportunities/topic-details/horizon-infra-2022-serv-01-01, accessed 10.10.2022.

^3^For more visit the toolkit at: https://ebrainsethicsandsociety.tekno.dk/.
